# *Acrocomia aculeata* oil-loaded nanoemulsion: development, anti-inflammatory properties, and cytotoxicity evaluation

**DOI:** 10.3762/bjnano.16.93

**Published:** 2025-08-06

**Authors:** Verónica Bautista-Robles, Hady Keita, Edgar Julián Paredes Gamero, Layna Tayná Brito Leite, Jessica de Araújo Isaías Muller, Mônica Cristina Toffoli Kadri, Ariadna Lafourcade Prada, Jesús Rafael Rodríguez Amado

**Affiliations:** 1 College of Pharmaceutical Sciences, Food and Nutrition. Federal University of Mato Grosso do Sul, Av. Costa e Silva s/n, 79070-900, Campo Grande-MS, Brazil; 2 Postgraduate Studies Division, University of Sierra Sur, Guillermo Rojas Mijangos s/n, 70800 Miahuatlán de Porfirio Díaz, Oaxaca, México; 3 Laboratory of Cellular and Molecular Biology, Faculty of Pharmaceutical Sciences, Food and Nutrition, Federal University of Mato Grosso do Sul, Av. Costa e Silva s/n, 79070-900, Campo Grande-MS, Brazil; 4 Laboratory of Pharmaceutical Technology, Federal University of Mato Grosso do Sul, Av. Costa e Silva, s/n, 79070-900, Campo Grande-MS, Brazil; 5 Laboratory of Pharmacology and Inflammation, Federal University of Mato Grosso do Sul, Av. Costa e Silva, s/n, 79070-900, Campo Grande-MS, Brazil; 6 Postgraduate Program in Health Sciences, Faculty of Health Sciences, Federal University of Grande Dourados, Dourados 79804-970, MS, Brazil

**Keywords:** *Acrocomia aculeata*, cytotoxicity, hemolysis, inflammation, nanoemulsion

## Abstract

The oil from the pulp of the bocaiúva fruit may have several medical applications. However, little is known about its pharmacological activity. Therefore, this study aimed to develop and evaluate the anti-inflammatory activity of a nanoemulsion loaded with the oil extracted from the pulp of the fruit of *Acrocomia aculeata*. Griffin’s method determined the hydrophilic–lipophilic equilibrium ratio of the nanoemulsion. It was shown to have an adequate droplet size (173.60 nm) with excellent homogeneity (polydispersity index 0.200). The anti-inflammatory activity of the nanoemulsion was evaluated by the carrageenan-induced paw edema method. Finally, the non-hemolytic and cytotoxic activity of the nanoformulation was determined to assess its safety. The nanoemulsion loaded with *Acrocomia aculeata* fruit pulp oil was shown to have parameters suitable for its characterization, impressive anti-inflammatory activity, and a safe profile.

## Introduction

*Acrocomia aculeata* Jacq is a palm of the Arecaceae family, commonly known as bocaiúva or macaúba. It is widespread in South America and is particularly abundant in Mato Grosso do Sul, located in the Center-West region of Brazil. *A. aculeata* stands out for its wide geographic distribution, being native to tropical forests [1–2]. Its rounded fruits present sensory attractions, such as color, distinctive and intense flavor, and aroma. They are traditionally consumed by the native population, occupying an important place in the regional culture [3–4].

Bocaiúva oil contains several antioxidant compounds such as phenols, terpenes, β-carotenes, and compounds that present antioxidant properties [5–6]. It contains free fatty acids, monoglycerides, triglycerides, sterols [6–8], and saturated and unsaturated fatty acids predominantly [9]. These compounds have the potential to enhance immune response, reduce the risk of degenerative diseases, and contribute to anti-inflammatory activity [10–11], reducing the indiscriminate use of non-steroidal anti-inflammatory drugs (NSAIDs) and corticosteroids in the population [12–13]. There are several reports about the severe adverse reactions in patients taking these drugs [14–15]. In addition, the economic impact of these degenerative and inflammatory diseases, such as rheumatoid arthritis, is significant, as demonstrated by heightened healthcare resource utilization and substantially increased annual aggregate costs. This underscores the need to find effective alternative treatments to prevent and treat these pathologies [16–18].

On a global scale, vegetable oils play a fundamental role not only in human nutrition but also as strategic inputs in the chemical, pharmaceutical, and food industries. In recent decades, there has been a growing imbalance between the demand and supply of these oils, which has generated challenges in terms of sustainability and supply. In this scenario, several oil palm species have been identified as potentially efficient sources of vegetable oil production, given their high yield rates per hectare and their ability to adapt to different agroecological environments [19]. In the state of Mato Grosso do Sul, it is possible to find virgin, refined, or conventional bocaiúva oil in the market for cosmetic, food, pharmaceutical, nutraceutical, and industrial applications. However, due to the chemical and physicochemical characteristics as well as the solubility and stability of this oil, it was decided to make a nanoemulsion to enhance its already known therapeutic benefits.

Nanoemulsions are nanoemulsified systems, either oil-in-water (O/W) or water-in-oil (W/O), which are isotropic, homogeneous, and thermodynamically unstable, with droplet sizes ranging from 20 to 200 nm [20]. They present properties such as high surface area per unit volume, robust kinetic stability, and tunable rheology [21]. It has been demonstrated that emulsified systems loaded with plant extracts have better pharmacological activity than extracts when used naturally [22]. For example, plant oil-loaded nanoemulsions exhibit high water solubility, improved permeability, and enhanced bioavailability [23]. This contrasts with the limited solubility and poor bioavailability of natural oil through different routes of administration [24–27].

The possibility of developing nanotechnological products with potent pharmacological activity was considered to add more value to the oil obtained from bocaiúva pulp, which contains phenols and carotenoids. Therefore, the objective of this study was to develop, characterize, and evaluate the anti-inflammatory activity of the nanoemulsion loaded with *Acrocomia aculeata* oil.

## Results and Discussion

Vegetable oils are known for their high content of fatty acids, which possess a diverse range of biological activities, including hypoglycemic [28], cholesterol-lowering, anti-inflammatory, and antioxidant effects [11,22–30]. Bocaiúva oil is widely used to treat cardiovascular, inflammatory, and renal diseases [31–32].

In addition, one of the main characteristics of this oil is its orange color due to the presence of phenols and carotenoids, which were characterized in this study. These secondary metabolites are considered to have high antioxidant activity and provide high stability to the oil [5]. These metabolites have been shown to possess anti-inflammatory and immunostimulant properties [1,33].

### Physicochemical characterization of *Acrocomia aculeata* fruit pulp oil

The physicochemical parameters of bocaiúva oil, such as acidity index, iodine index, and refractive index, were analyzed. The acidity index indicates the state of conservation of oils and fats and is related to the oxidation process. Our results showed an acid index of 0.92 ± 0.10. The iodine index determines the amount of unsaturation in fatty acids [28]. Our results showed an iodine index of 74.50 ± 1.50 g I_2_/100 g, values that are within the range allowed (58–75 g I_2_/100 g) by OMS/FAO for oils with high oleic acid content [34].

Also, quality indicators such as refractive index, solubility in different organic solvents, and relative density showed that the bocaiúva oil used in that study had good purity [35]. Coimbra and Jorge analyzed *Acrocomia aculeata* oil and found refractive index values similar to those in this study (1.46 ± 0.01) [19]. These results were found within the reference values established for oils rich in oleic acids, such as extra virgin olive oil, palm oil, and almond oil [34]. The presence of polyphenols and carotenoids was also identified in this oil (see Table 1).

**Table 1:** Physicochemical properties of *Acrocomia aculeata* fruit pulp oil.

Property	Value

relative density	0.9000 ± 0.0001
iodine value (g I_2_/100 g)	74.50 ± 1.50
refractive index (30 °C)	1.456 ± 0.001
peroxide value (mEq/kg oil)	4.50 ± 0.40
saponification index (mg KOH/g)	133.00 ± 4.50
acidity	0.92 ± 0.10
total carotenoids (µg/g)	266.00 ± 12.00
polyphenols (mg/g)	12.60 ± 0.30

Table 2 shows the profile of fatty acids present in *Acrocomia aculeata* fruit pulp oil. Oleic acid is the major component (71.25%) among monounsaturated fatty acids (73.79%). Therefore, bocaiúva oil can be considered an oil with a cardioprotective effect due to its high oleic acid content [36–37]. In addition, its levels of monounsaturated fatty acids are higher than those found in extra virgin olive, soybean, corn, sunflower, and flaxseed oils [37–38].

**Table 2:** Lipid profile of *Acrocomia aculeata* fruit pulp oil.

Fatty acid	Content (%)	RE Index	LR^a^ Index

saturated			

hexanoic acid	0.22 ± 0.02	974	975
octanoic acid	0.25 ± 0.02	1169	1170
decanoic acid	0.13 ± 0.01	1365	1365
dodecanoic acid	0.85 ± 0.01	1548	1547
tetradecanoic acid	0.70 ± 0.01	1747	1749
hexadecanoic acid	16.52 ± 0.15	1970	1969
octadecanoic acid	4.11 ± 0.15	2164	2165
docosanoic acid	0.06 ± 0.03	2562	2564
subtotal	22.84 ± 0.05	—	—

monounsaturated			

9-hexadecenoic acid	2.54 ± 0.01	1939	1938
9-octadecenoic acid	71.25 ± 2.21	2241	2142
subtotal	73.79 ± 1.11	—	—

polyunsaturated			

9,12,15-octadecatrienoic acid	0.80 ± 0.04	2154	2155
9,12-octadecadienoic acid	2.20 ± 0.33	2176	2175
eicosanoic acid	0.20 ± 0.03	2369	2370
subtotal	3.20 ± 0.13	—	—
total fatty acids	>99.00%	—	—

^a^Literate retention rate (from NIST chemistry webbook, SRD 69).

The bocaiúva oil utilized in this study demonstrated excellent quality, as assessed by established parameters for evaluating vegetable oils reported in the literature [11,39]. The results are consistent with findings by Hiane and collaborators [40] and Lieb and collaborators [7], who also observed a high concentration of monounsaturated fatty acids in the fruit pulp. Amaral et al. further identified a notable oleic acid content of 69.07% in *Acrocomia aculeata* pulp oil [41]. Minor discrepancies in composition may be attributed to variations in environmental conditions, such as climatic conditions, temperature, and pulp drying duration before oil extraction. Additionally, the specific extraction technique employed can influence lipid degradation and promote the formation of free fatty acids [19]. Despite these variations, the compositional profiles remain comparable, underscoring the distinctive chemical characteristics of the oil studied.

### Preparation of nanoemulsions, required hydrophilic–lipophilic balance, droplet size, zeta potential, and shelf stability

The development of a nanoemulsion requires the determination of key formulation parameters, including the required hydrophilic–lipophilic balance (HLBr), droplet size, and polydispersity index (PDI) [42–44]. In this study, the *Acrocomia aculeata* oil nanoemulsion (AANE) exhibited a uniform droplet size distribution and a stable PDI upon formulation with the surfactant system characterized by a hydrophilic–lipophilic balance (HLB) value of 12. These physicochemical parameters are critical for defining the kinetic stability and structural integrity of the nanoemulsion system.

**Figure 1:**
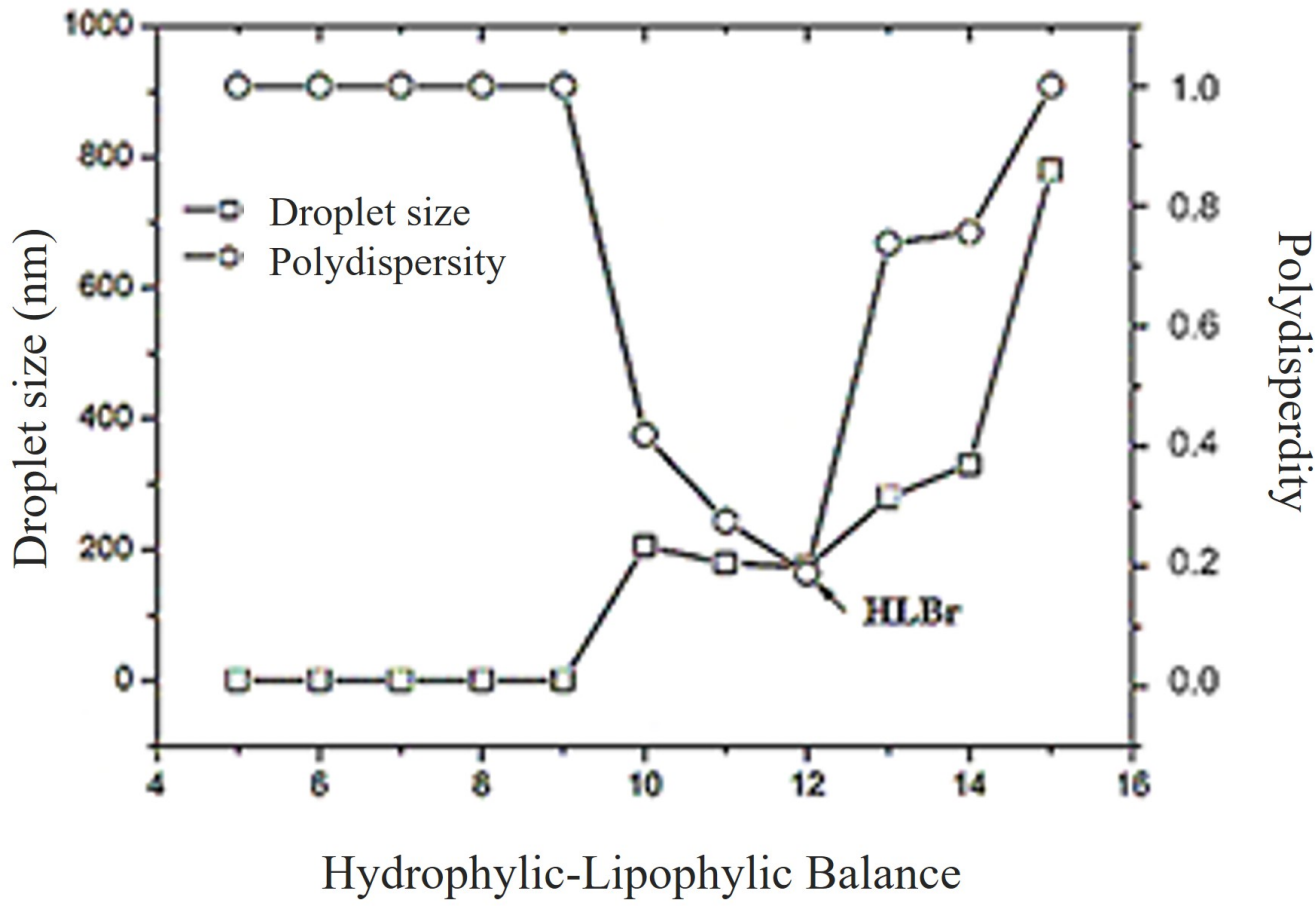
Droplet size and polydispersity index of nanoemulsions versus the hydrophilic–lipophilic balance of the surfactant system used for the preparation. HLBr: required hydrophilic-lipophilic balance.

Surfactants or emulsifiers are characterized by their HLB values, which reflect their affinity for either aqueous or lipid phases. Hydrophilic emulsifiers typically exhibit a high HLB value, while lipophilic emulsifiers possess lower values. The HLB scale generally ranges from 1 to 20, with an approximate midpoint of 10, distinguishing emulsifiers suited for oil-in-water versus water-in-oil systems [43] (Figure 1).

**Figure 2:**
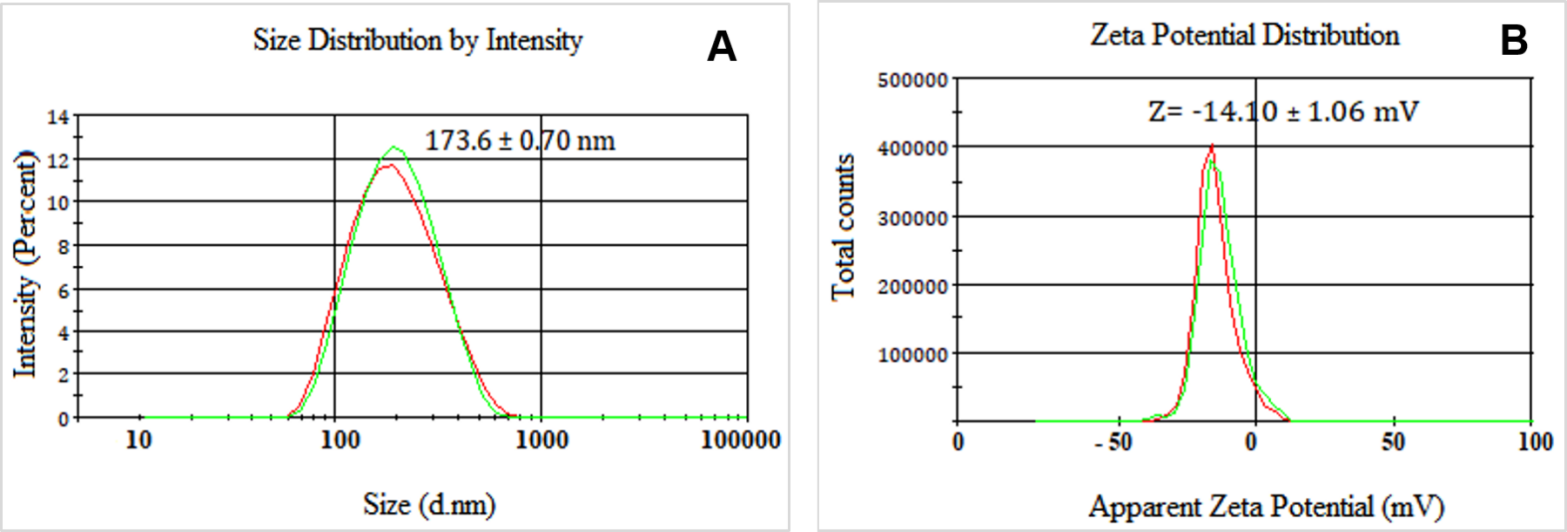
Droplet size distribution and zeta potential of the nanoemulsion prepared with 0.28 parts Span 80**^®^** and 0.72 parts Tween 80**^®^** (HLB = 12). Droplet size: 173.6 ± 0.70 nm. Zeta potential: 14.0 ± 1.06 mV.

A surfactant system characterized by an HLB value of 12 (Figure 1) was employed to formulate the bocaiúva oil nanoemulsion, resulting in a satisfactory polydispersity index of 0.200. The formulation exhibited excellent physical stability, maintaining consistent zeta potential and droplet size parameters over a 180-day storage period at 25 ± 2 °C. Dynamic light scattering analysis revealed a mean nanodroplet size (by intensity) of 173.6 ± 0.70 nm (Figure 2A). The nanoemulsion, composed of 0.28 parts of Span 80**^®^** and 0.72 parts of Tween 80**^®^,** exhibited a zeta potential of −14.10 ± 1.06 mV (Figure 2B), indicative of sufficient electrostatic repulsion for colloidal stability.

It should be noted that the phenolic compounds and carotenoids contained in this oil are considered potent antioxidants, which may contribute to the stability of the nanoemulsion [45–46].

**Figure 3:**
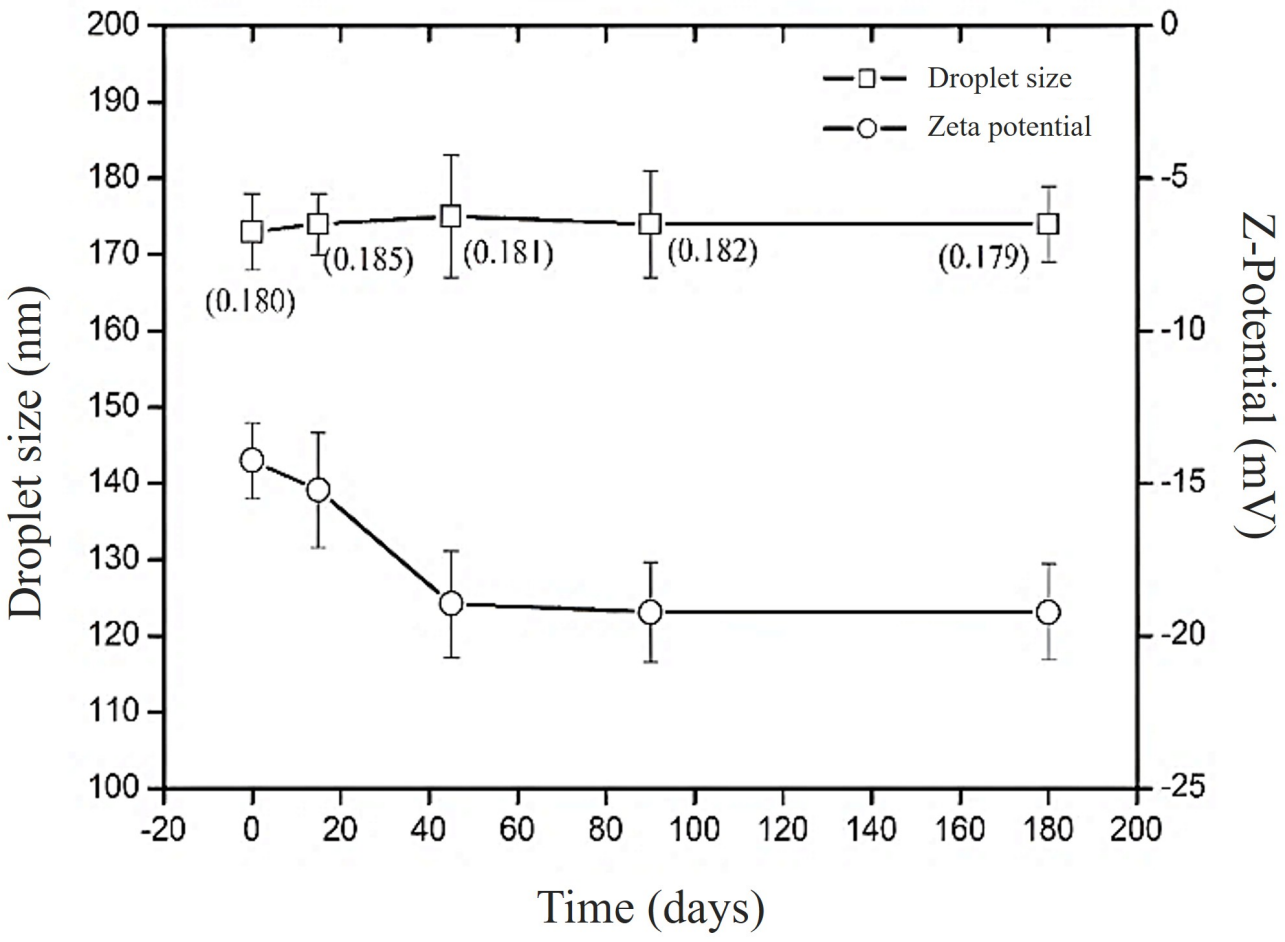
Stability of bocaiúva oil-loaded nanoemulsion prepared with 0.28 parts of Span 80**^®^** and 0.72 parts of Tween 80**^®^** (HLB = 12).

Figure 3 shows the behavior of zeta potential and droplet size in Bocaiúva oil-loaded nanoemulsion over 180 days. The droplet size remained stable at around 170 nm, and no statistical differences were found at any point in time over 180 days (*F*_test_ = 0.18, *p*_value_ = 0.0804). In contrast, the zeta potential underwent a significant decrease from approximately −10 to −20 mV within the first 45 days and then stabilized for the remainder of the 180 days. The analysis of variance found statistically significant differences among the zeta potential values (*F*_test_ = 2.4258, *p*_value_ = 0.0021). The Tukey test suggests that the zeta potential values at zero and 15 days were not statistically different, forming a homogenous group statistically different from the rest of the time points (45, 90, and 180 days), which formed another homogeneous group.

The progressive increase in the absolute value of zeta potential over time suggests enhanced system stability, attributed to the intensification of electrostatic repulsion among nanodroplets. Although this phenomenon is atypical for systems stabilized by nonionic surfactants (Span 80**^®^** and Tween 80**^®^**), it may result from the presence of ionizable bioactive compounds such as phenolics and carotenoids, which can associate with the droplet interface. These compounds likely expand the diffuse electrical double layer surrounding the nanodroplets, thereby increasing the magnitude of the zeta potential, enhancing stability by preventing droplet aggregation [47].

**Figure 4:**
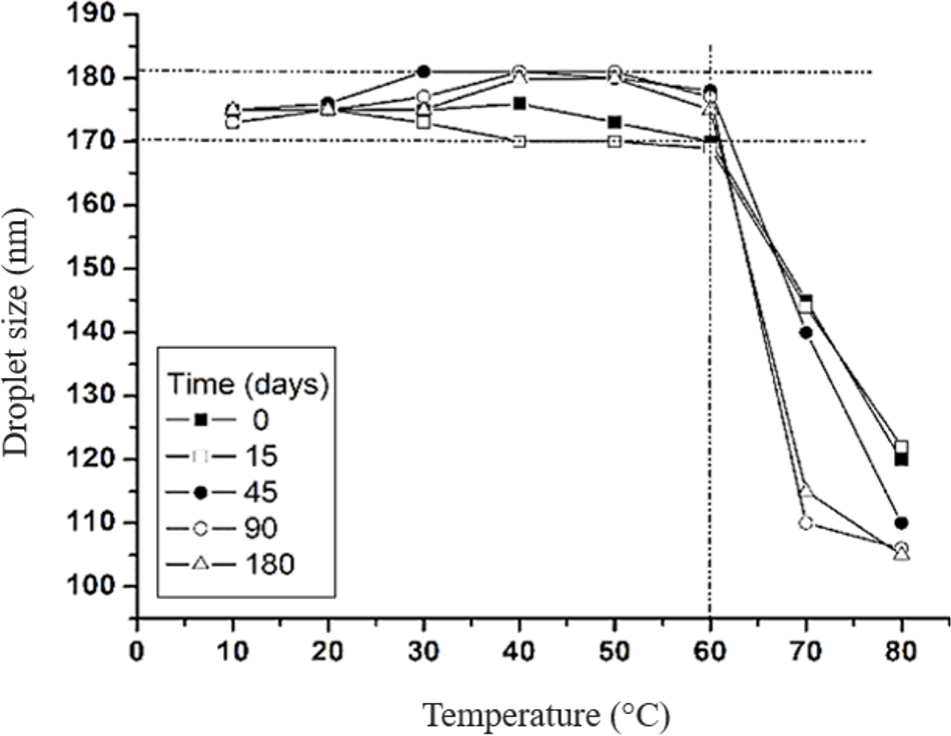
Effect of temperature on droplet size of nanoemulsion loaded with bocaiúva oil.

Figure 4 presents the impact of temperature (ranging from 10 to 80 °C) on the nanodroplet size of the AANE formulation over a 180-day period. Between 10 and 60 °C, the nanodroplet size remained relatively stable, ranging from 171 to 181 nm. However, temperatures exceeding 60 °C led to a marked reduction in droplet size, stabilizing between 110 and 120 nm across all time points. This reduction can be attributed to several interrelated physicochemical mechanisms. Primarily, the nonionic surfactants Span 80^®^ and Tween 80^®^ reduce interfacial tension between oil and aqueous phases, a phenomenon that becomes increasingly efficient at elevated temperatures, promoting the formation of smaller droplets. Additionally, elevated temperatures enhance molecular mobility and solubility, facilitating droplet disruption and dispersion effects observed in bocaiúva oil nanoemulsions above 60 °C [48]. Moreover, the surfactants exhibit temperature-responsive behavior, reorganizing at higher temperatures to stabilize finer dispersions. The concurrent decline in oil phase viscosity with increasing temperature also improves emulsification efficiency by promoting shear-induced droplet breakup, particularly under mechanical agitation [48]. Nevertheless, while these conditions favor the formation of smaller nanodroplets, temperatures above 60 °C may compromise emulsion stability, potentially triggering phase separation or degradation of labile components. This highlights the necessity for stringent temperature control during both formulation and storage to ensure the long-term stability of the nanoemulsion [49].

### Hemolytic and cytotoxic activity of *Acrocomia aculeata* oil-based nanoemulsion

**Figure 5:**
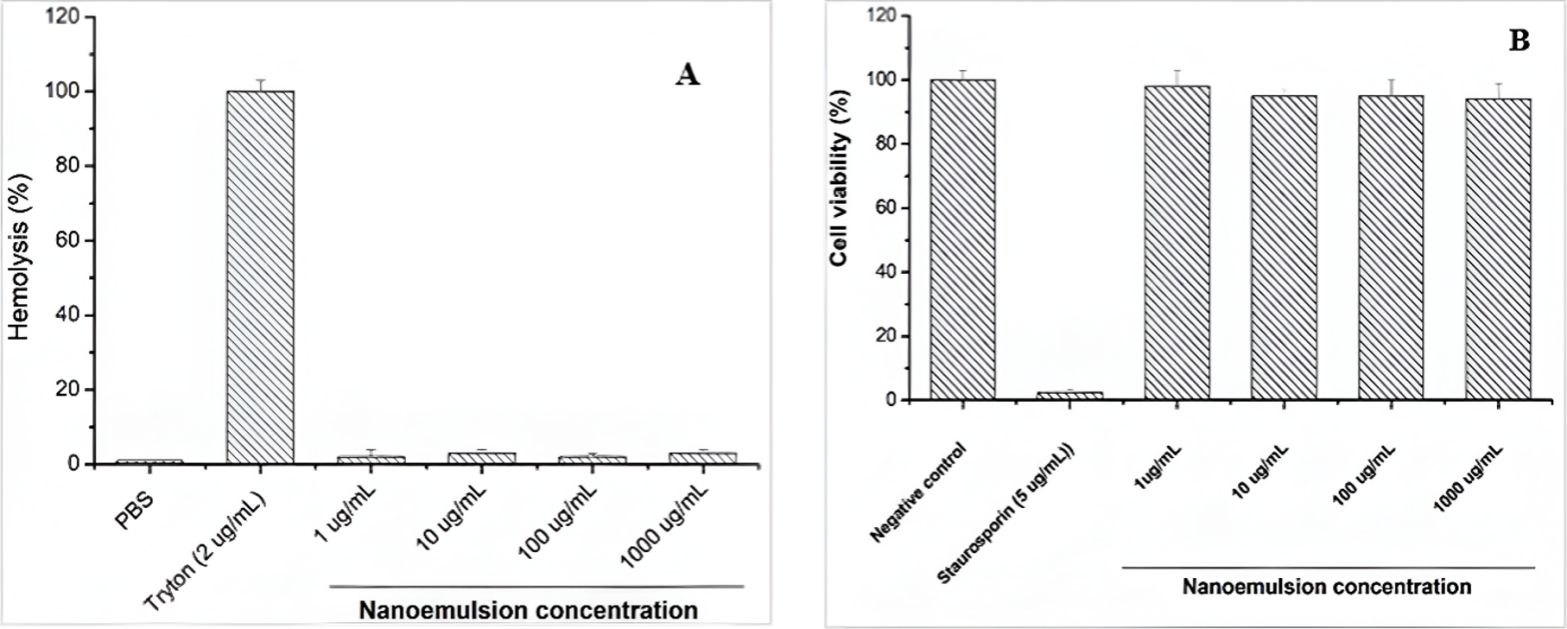
Hemolytic effect (A) and cell viability assay (B) of nanoemulsion loaded with *Acrocomia aculeata* fruit oil.

The hemolytic and cytotoxic activities of the nanoemulsion were assessed to evaluate its potential therapeutic use, with particular focus on its interaction with erythrocyte membranes. The nanoemulsion demonstrated no hemolytic activity against murine erythrocytes at concentrations of 1, 10, 100, and 1000 μg/mL. These findings were benchmarked against Triton X-100, a well-established positive control known for its potent hemolytic effect [50]. As shown in Figure 5A, AANE maintained erythrocyte membrane integrity across all tested concentrations, reinforcing its biocompatibility.

Furthermore, cytotoxicity evaluation showed that AANE did not inhibit cell growth, as cellular viability remained at 100% across all tested concentrations (1, 10, 100, and 1000 μg/mL, Figure 5B). These findings underscore the nanoemulsion’s biocompatibility and support its potential for safe therapeutic applications. This is consistent with the traditional use of bocaiúva oil, which is commonly taken in or applied topically by traditional populations for managing joint inflammation and some infections [51]. Nevertheless, further comprehensive studies are necessary to confirm the long-term safety and therapeutic viability of the bocaiúva oil loaded nanoemulsions.

### Anti-inflammatory activity of *Acrocomia aculeata* oil-based nanoemulsion

After confirmation of the nonhemolytic effect of the nanoemulsion, the anti-inflammatory effect was evaluated. In inflammatory processes, therapeutic interventions aim primarily to attenuate the productive phase of inflammation, particularly by inhibiting leukocyte infiltration at the injury site [52].

In this study, acute inflammation was induced via subplantar injection of carrageenan, a sulfated polysaccharide known to stimulate edema through the release of pro-inflammatory mediators associated with hyperalgesia and vascular alterations [53–54]. The paw edema model provides a reliable assessment of two key inflammatory parameters, namely, leukocyte migration and protein extravasation [55].

**Figure 6:**
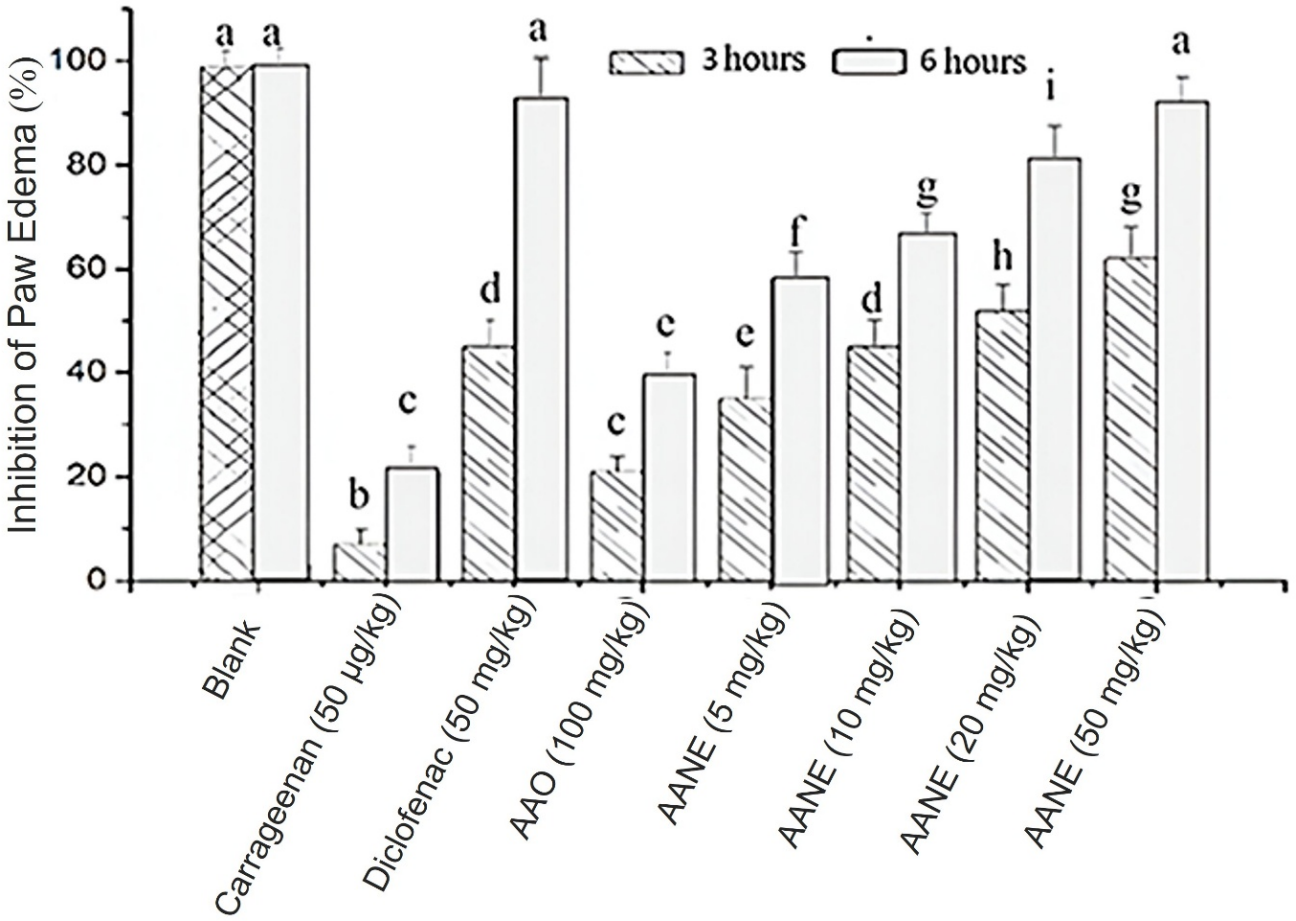
Anti-inflammatory effect of *Acrocomia aculeata* fruit oil and oil-loaded nanoemulsion. Different letters indicate statistically significant differences at *p* ≤ 0.05.

The assay demonstrated that *Acrocomia aculeata* nanoemulsion at a dose of 50 mg/kg has a pharmacological effect approximately two-fold greater than that of the pristine oil at 100 mg/kg (Figure 6). This finding reinforces the premise that the nanoformulation of drugs potentially enhances the biological activity of natural drugs [22]. Similar outcomes were reported by Silva et al., who observed enhanced anti-inflammatory efficacy of a *Copaifera spp*. essential oil nanoemulsion compared to its unformulated counterpart [47].

The superior pharmacological response of the nanoemulsion may be attributed to the nanoscale droplet size, which increases the surface area-to-volume ratio, enhances solubility and stability, and promotes rapid absorption and cellular uptake [56]. The nanometric scale facilitates more efficient interaction with cellular receptors, contributing to its heightened bioactivity [57]. Collectively, these findings suggest that bocaiúva oil-based nanoemulsions hold promising potential as anti-inflammatory agents.

Further analysis using the carrageenan-induced paw edema model revealed that the oil at 100 mg/kg exerted a modest anti-inflammatory effect up to 3 h post-treatment, which was considerably lower than that of both diclofenac and the nanoemulsion at 5, 10, 20, and 50 mg/kg body weight. Notably, at the 3 h time point, the nanoemulsion at 20 and 50 mg/kg elicited a more potent anti-inflammatory response than diclofenac. At 6 h post-treatment, the nanoemulsion maintained a comparable effect to diclofenac at the same dose levels (Figure 6). These results further substantiate the enhanced efficacy of the oil in the form of nanoemulsion in modulating acute inflammation in rats.

## Conclusion

The *Acrocomia aculeata* oil-loaded nanoemulsion exhibited a homogeneously distributed droplet size (173.60 nm) within the nanometric range and demonstrated excellent physicochemical stability, maintaining its structural integrity and key parameters over a six-month period of shelf storage. Nanoemulsion showed a markedly greater anti-inflammatory effect compared to unformulated bocaiúva oil with an efficacy comparable to that of diclofenac. In addition, this nanoemulsion showed no cytotoxicity or hemolytic activity, indicating a favorable safety profile. These findings underscore the potential of the *Acrocomia aculeata* oil-loaded nanoemulsion as a nanotechnological innovative product that enhances the therapeutic value of *A. aculeata* oil and supports its development as a promising anti-inflammatory agent.

## Experimental

### Materials

Bocaiúva oil was used as the lipid core (relative density 0.9000, iodine value 74.50 g I_2_/100 g, refractive index (30 °C): 1.456, peroxide value 4.50 mEq/kg, saponification index: 133.00 mg KOH/g ± 4.50) characterized in this study. The food-grade surfactant was Tween^®^ 80 (nonionic polyoxyethylene (20) sorbitan monooleate; C_64_H_124_O_26_; HLB = 15.0), Span 80^®^ (2*R*)-2-[(2*R*,3*R*,4*S*)-3,4-dihydroxyoxyoxolan-2-yl]-2-hydroxyethyl (9*Z*)-octadec-9-enoate; C_24_H_44_O_6_ HLB 4.3). Deionized water was used in the preparation of all experiments throughout the study.

### Plant material

The fruits of *Acrocomia aculeata* were collected in Campo Grande, Mato Grosso do Sul, Brazil (20°50'00.1" S 54°36'45.7" W) after the natural fall of the first ripe fruits. The fruit pulp was manually separated from the seeds and preserved until oil extraction.

### Bocaiúva oil extraction

One kilogram of fresh fruit pulp was placed in an Erlenmeyer flask and extracted with *n*-hexane (1000 mL) by mechanical agitation for 24 h. The *n*-hexane solution was separated from the pulp and preserved. Another 500 mL of *n*-hexane was added to the pulp for a second extraction under the same conditions. The two extractions were combined in a rotary evaporator system (Ika Werke, Germany). It was subjected to a slow stream of nitrogen for 24 h to obtain the solvent-free oil.

### Physicochemical characterization of *Acrocomia aculeata* oil

Relative density and refractive index of *Acrocomia aculeata* oil (AAO) were evaluated according to the American Pharmacopoeia [58]. Iodine value, peroxide value, acid value, and saponification index were also evaluated following the protocols of the Brazilian Pharmacopoeia [58].

### Determination of phenolic content of *Acrocomia aculeata* oil

The total phenols present in AAO were evaluated using the Folin–Ciocalteu spectrophotometric method. In this method, 3 mL of bocaiúva oil are mixed with 10 mL of a 75% ethanol solution. The mixture was stirred on a mechanical shaker for 2 h and allowed to stand in the dark for 24 h. The liquid was then centrifuged at 5000 rpm (LKP, Brazil). Aliquots of 1 mL of the ethanolic phase were used for analysis. The calibration curve was constructed using the standard addition method and a standard reference material (Sigma, USA). The results were expressed as gallic acid equivalent.

### Determination of carotenoid content

Carotenoid content was evaluated spectrophotometrically (Shimatsu, Japan) following the procedure described by Rodriguez-Amaya. The molar extinction coefficient of β-carotene (β-C) in *n*-hexane at 453 nm (2592 mol^−1^·cm^−1^) was used. The carotenoid content (CT), expressed as β-carotene, was calculated by the formula:


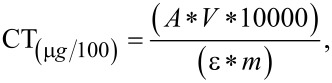


where *A* is the absorbance of the sample, *V* is the volume of the sample, ε is the molar absorbance of β-carotene in *n*-hexane at 453 nm, and *m* is the mass of the sample [59].

### Lipid profile

A derivatization process was carried out to improve the stability of bocaiúva samples. One gram of bocaiúva oil was dissolved in *n*-hexane and vortexed for 5 min. The hexane phase was separated by centrifugation, transferred to a derivatization tube, and dried under a stream of nitrogen for 24 h. Then, 3 mL of a 2% methanolic NaOH solution was added to the tube. The tube was hermetically sealed and heated at 85 °C for 3 min. After cooling to room temperature, 2 mL of a BF_3_/methanol solution was added. The tube was resealed and heated for 25 min.

Once cooled, the solution was extracted with 5 mL of *n*-hexane and centrifuged. 20 μL of supernatant (hexane phase) were injected directly into the GC-MS system (Mega 2 series gas chromatograph coupled to a SHIMADZU GC-MS-QP500 mass spectrometer (GC-MS) (Japan)) [60]. A 30 m × 0.32 mm capillary column with a 0.25 mm thick layer (66DB-5MS, Agilent Technologies, USA) was used as the stationary phase. Helium gas was used as carrier gas at a flow rate of 1.0 mL/min with a split ratio of 1:10. The injector temperature was set to 250 °C. The oven temperature was set to 130 °C for 10 min and then increased to 250 °C at a rate of 5 K/min, maintaining the final temperature for 10 min.

Mass spectra were acquired using a mass range of *m*/*z* 40–500, an interface temperature of 250 °C, and an ion source temperature of 220 °C. The solvent cutoff time was 3 min, and the event time was 0.20 min. The sweep speed was set at 2,500 mL/min. The composition (in percent) was calculated using the peak normalization method.

### Preparation of nanoemulsions

*Acrocomia aculeata* oil nanoemulsions were prepared using the phase inversion method [61–62]. The formulations comprised 5% w/w bocaiúva oil, 5% surfactants (Span 80**^®^**: Tween 80**^®^**), and 90% deionized water. The organic phase, composed of bocaiúva oil and surfactants, was stirred at 400 rpm at 35 °C for 20 min. The aqueous phase (deionized water with conductivity below 0.4 μS and pH 6.5) was added to the organic phase at 1 mL/min under continuous magnetic stirring (400 rpm). Stirring was maintained for 20 min after adding the total volume of water. Finally, the initial volume of the nanoemulsion (50 mL) was restored with deionized water [42].

### Required hydrophilic–lipophilic balance (HLBr)

Griffin's method determined the hydrophilic–lipophilic balance (HLBr) necessary to emulsify bocaiúva oil [63]. A set of nanoemulsions was prepared using HLB values from 4.3 to 15, obtained by mixing different proportions of Span 80**^®^** (HLB 4.3) and Tween 80**^®^** (HLB 15). The temperature was maintained at 25 ± 1 °C. The surfactant mixture that produced the stable nanoemulsion with the smallest droplet size was selected as the (HLBr) to emulsify bocaiúva oil [63].

### Droplet size and zeta potential

Droplet size and polydispersity index (PDI) were measured by dynamic light scattering (DLS) with a Zetasizer Nano-ZS instrument (Malvern, UK). Zeta potential was determined by electrophoretic light scattering with a Zetasizer Nano-ZS instrument (Malvern, UK). AANE was diluted with Milli-Q water (1:25, v/v). All measurements were performed in triplicate, and results were presented as the mean ± standard deviation [42].

### Shelf stability

The selected AANE was transferred to an amber vial and stored at 25 ± 2 °C for 180 days. Droplet size, polydispersity index, and zeta potential were measured at 0, 15, 45, 90, and 180 days. Measurements were performed in triplicate, and results were presented as the mean ± standard deviation.

The effect of temperature on droplet size was also evaluated, from 10 to 70 °C, at the same time intervals mentioned above. Measurements were performed with the Zetasizer instrument (Malvern, UK). The nanoemulsion was equilibrated at temperatures of 10, 20, 30 40, 40, 50, 60, and 70 °C for 5 min prior to measurement [42].

### Hemolytic activity

Hemolytic activity was assessed using a murine erythrocyte suspension as described by Amado et al. Briefly, 190 μL of erythrocyte suspension was added to the wells of a 96-well polycarbonate plate. Then, 10 μL of nanoemulsion solution at different concentrations in PBS buffer (0, 5, 10, 20, and 50 μg/mL) was added to each well [41]. The plates were incubated for 1 h at 37 °C and were centrifuged at 3000 rpm at 5 °C for 15 min. After centrifugation, the concentration was suspended in 50 mL of phosphate-buffered saline (PBS, pH 7.4). The amount of hemoglobin was determined at 540 nm using a docetaxel (DTX) 880 multi-mode detector (Beckman, UK). A solution of 10 μg/mL Triton X-100 was used as a positive control, and 10 μL of PBS was used as a negative control. The assay was performed in triplicate.

The hemolytic activity (% of Hemolysis) was calculated using:


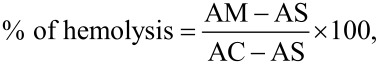


where AM is the absorbance of the sample; AS is the absorbance of the solvent, and AC is the absorbance of the positive control [61–62].

### Cytotoxic activity

The cytotoxic activity of AANE was evaluated in murine macrophages of the J774 strain (ATCC USA) according to the technique described by Nakayama and colleagues [64]. Staurosporine (5 μg/mL) was used as a positive control, whereas cells from the culture without the nanoemulsion served as a negative control. Different concentrations of the nanoemulsion (1.65, 3.30, 6.60, 12.5, 25, 50, and 100 μg/mL) were added to the cultured cells and kept in contact for 24 h. Assays were performed in triplicate, and cell viability was expressed as a percentage according to International Organization for Standardization ISO 10993-5 guidelines [65].

### Anti-inflammatory activity of *Acrocomia aculeata* oil-based nanoemulsion

#### Animals

The anti-inflammatory effect was evaluated using carrageenan-induced paw edema. Six- to eight-week-old female Swiss mice weighing 22 to 28 g were used [66]. Animals were acclimatized under laboratory conditions (25 ± 3 °C, 65 ± 5% humidity) with a 12/12 h light/dark cycle. Animals had free access to food and water at all times and were deprived of food 6 h before the experiment.

#### Formation of experimental groups and induction of paw edema

Eight experimental groups were randomly formed, with five animals per group (*n* = 5). 30 min before edema induction, groups 3, 4, 5, 6, 7, and 8 received the test substances (diclofenac sodium, AAO, or AANE). Group 1 received a blank (obtained under the same conditions, but without AAO) and group 2 received only carrageenan as it is explained in Table 3.

Therefore, the experimental groups were organized as follows:

- Group 1 recieved a blank (i.e., no carrageenan) but AANE treatment.
- Group 2 received only water before carrageenan administration.
- Group 3 received diclofenac sodium before carrageenan administration.
- Group 4 received AAO before carrageenan administration.
- Groups 5, 6, 7, and 8 received AANE before carrageenan administration.

**Figure 7:**
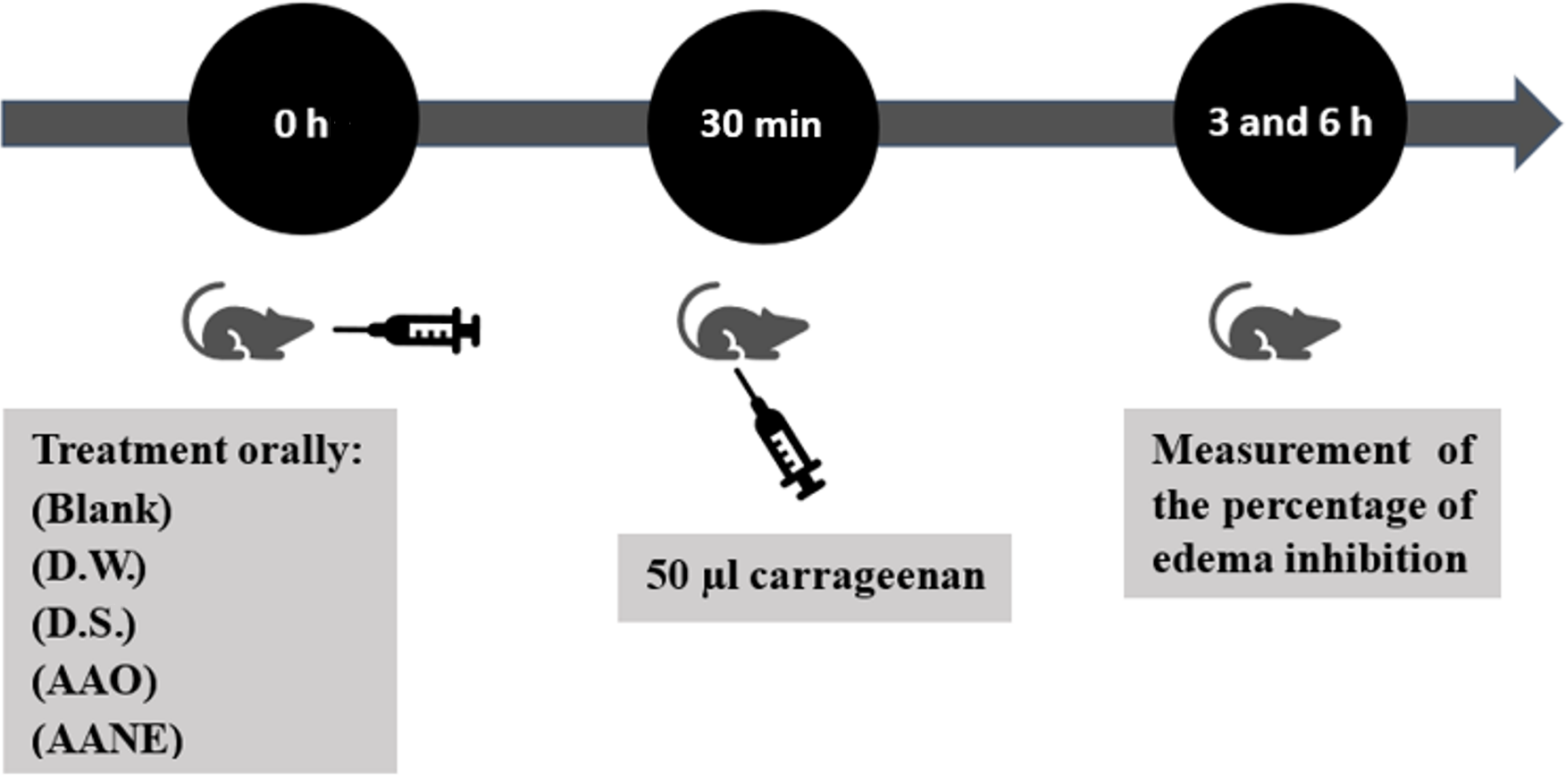
Representative scheme of the experimental protocol of paw edema induced by carrageenan. D.W. = distilled water; D.S. = diclofenac sodium; AAO = *Acrocomia aculeata* oil; AANE = *Acrocomia aculeata* oil nanoemulsions.

Table 3 details how the experiment (Figure 7) was conducted with the corresponding doses of each treatment.

**Table 3:** Experimental groups of the paw edema protocol induced by carrageenan.

Groups	Treatment orally	Induction of edema

Group 1 (blank) (mg/kg body weight)	50	—^a^
Group 2 (D.W.^b^) (µL)	200	50 µL carrageenan
Group 3 (D.S.^c^) (mg/kg body weight)	50	50 µL carrageenan
Group 4 (AAO^d^) (mg/kg body weight)	100	50 µL carrageenan
Group 5 (AANE^e^) (mg/kg body weight)	5	50 µL carrageenan
Group 6 (AANE^e^) (mg/kg body weight)	10	50 µL carrageenan
Group 7 (AANE^e^) (mg/kg body weight)	20	50 µL carrageenan
Group 8 (AANE^e^) (mg/kg body weight)	50	50 µL carrageenan

^a^50 µL injected saline; ^b^D.W. = distilled water; ^c^D.S. = diclofenac sodium; ^d^AAO = *Acrocomia aculeata* oil; ^e^AANE: *Acrocomia aculeata* oil nanoemulsions.

Edema volume was measured by plethysmometry (NovaLab, Brazil) at 3 and 6 h after carrageenan injection [67]. Inhibition of paw edema was expressed (in percentage) as the difference between the control value (paw volume of each animal before carrageenan injection) and the volumes measured at each time point after the treatments [63].

The expression of the results obtained, which is calculated using the formula:


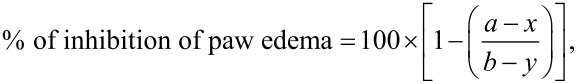


where *a* = mean hind paw volume of test/standard group animals after carageenan injection, *b* = mean hind paw volume of positive control animals after carageenan injection, *x* = mean hind paw volume of test/standard group animals before carageenan injection, *y* = mean hind paw volume of positive control animals before carageenan injection [51].

#### Statistical analysis

A one-way ANOVA followed by Tukey’s HSD test was performed to determine statistical differences between experimental groups. A statistically significant difference was considered at *p* ≤ 0.05. StatGraphics^®^ Centurion XV.1 software (StatEase, USA) was used for the analyses.

### Ethical approval

All experiments were performed in accordance with the Ethics Committee for the Experimental Use of Animals of the Federal University of Mato Grosso do Sul, Brazil (Reference number: 1.250/2022).

## Data Availability

Data generated and analyzed during this study is available from the corresponding author upon reasonable request.
